# Endoscopic treatment of esophagogastric and esophagojejunal anastomotic leaks: A single tertiary center experience

**DOI:** 10.1097/MD.0000000000035582

**Published:** 2023-10-13

**Authors:** Mustafa Cengiz, Bulent Odemis, Muhammed Bahattin Durak

**Affiliations:** a Gulhane Research and Training Hospital, Department of Gastroenterology, Etlik, Ankara, Turkey; b Ankara City Hospital, Department of Gastroenterology, Ankara, Turkey.

**Keywords:** anastomotic leak, cancer surgery, endoscopic treatment, fully covered self-expanding metallic stents

## Abstract

Anastomotic leakage in esophagogastric and esophagojejunal anastomoses after esophagectomy/gastrectomy is a severe complication with a high mortality rate. We aimed to evaluate the technical and clinical success and outcomes of endoscopic placement of fully covered self-expanding metallic stents (FCSEMSs) for treating anastomotic leaks that develop after cancer surgery. All consecutive patients treated at the Gastroenterology Department, Ankara City Hospital, Ankara, Turkey, who underwent endoscopic FCSEMSs for leaks of esophagogastric or esophagojejunal anastomosis between February 2015 and December 2021 were included in the study. We analyzed the data on leak characteristics, technical success, clinical success, stent-related complications, and mortality to investigate the clinical efficacy and safety of endoscopically implanted FCSEMSs. A total of 24 patients, 12 of whom were male were included in the study. The median age of the patients was 60 years (min-max: 38–84). Nineteen patients underwent esophagojejunal anastomosis, and 5 patients underwent esophagogastric anastomosis. The median stent follow-up time was 68.8 (26–190) days, and the median hospital stay was 62.7 (24–145) days. Complications related to stent placement were observed in of 50%. The most common complication was stent migration, occurring at a frequency of 37.5%. The median follow-up period time was 11.4 (2–37) months. While the clinical success rate was 87.5%, 3 patients died. Endoscopic placement of FCSEMSs is a relatively safe and beneficial treatment for esophagojejunal and esophagogastric anastomotic leaks.

## 1. Introduction

Gastrectomy and esophagectomy are commonly used procedures in patients with esophagogastric malignancies. Anastomotic leak is a severe complication that seen in 5% to 8% of gastrectomy and 35% to 25% of esophagectomies.^[1–3]^

As high rates of mortality may occur after revision surgery, various conservative and endoscopic procedures are being developed to reduce the mortality rate in these cases.^[2–4]^

The reoperation has a high morbidity and should be avoided unless conservative therapy has failed and the use of fully covered self-expandable metallic stents (FCSEMSs) as outlined in the literature simply adds to the armamentarium of several types of conservative therapy. The endoscopic placement of FCSEMSs has recently been considered an effective procedure for treating anastomotic leaks.^[5,6]^ Patients with esophageal leak have higher morbidity and mortality rates than those who recover without any complications.^[7–9]^ There is no consensus on the endoscopic management of esophageal leaks due to the resection of malignant lesions of the esophagus and stomach.

We aimed to evaluate the technical and clinical success and outcomes of endoscopic placement of FCSEMSs in the treatment of anastomotic leaks that develop after cancer surgery.

## 2. Methods

### 2.1. Patients

All consecutive patients treated at the Gastroenterology Department, Ankara City Hospital, Ankara, Turkey, who underwent endoscopic FCSEMS placement to treat postoperative leaks of esophagogastric or esophagojejunal anastomosis after cancer surgery between February 2015 and December 2021, were included in the study. Since the center where we work is the center where advanced endoscopic procedures are performed, we included patients with postoperative esophagogastric/esophagojejunal anastomotic leaks who were referred both from our surgery clinic and from many other centers. As we included the referred patients in the study, we could not naturally determine how many patients underwent esophagogastric/esophagojejeunal anastomosis in total.

### 2.2. Data collection

Data on the clinical, demographic, disease-related, and operation-related characteristics of the patients, size of the anastomotic leak, technical success, clinical success, and complications of FCSEMSs were collected retrospectively by a clinician and recorded in a database.

### 2.3. Procedural evaluation

**Figure 1.**
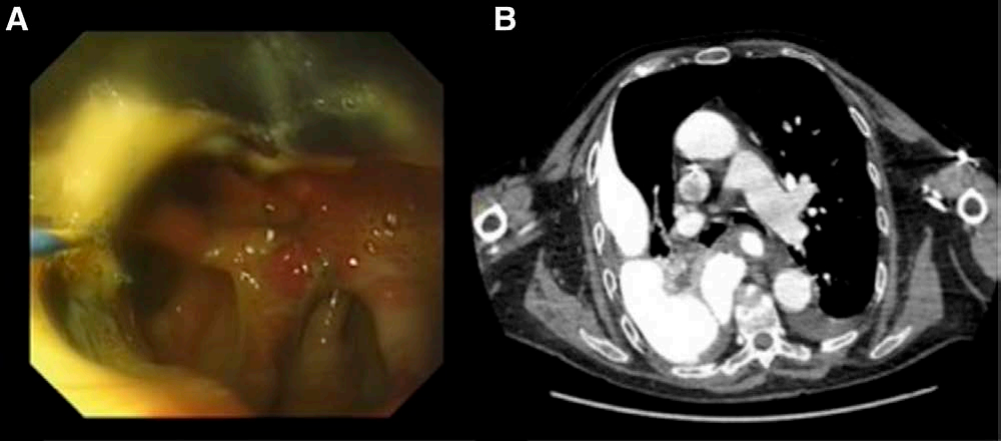
An endoscopic image (A) and a cross-sectional CT image (B) of an anastomotic leak. CT = computed tomography.

Anastomotic leak was diagnosed by endoscopy or visualization of perianastomotic contrast or air on thoraco-abdominal computed tomography (CT) using oral contrast material. When the separation in the anastomosis line could not be clearly evaluated with an endoscope, the leak site was identified using methylene blue administered through a percutaneous drain. Anastomotic leak was categorized according to the Carboni classification: class I, no dehiscence; class II, <10% dehiscence; class III: 10% to 50% dehiscence; and class IV, more than 50% dehiscence.^[10]^ Since only patients with anastomotic leak were included in this study, patients with conduit necrosis were excluded. In addition, staple lines of gastric conduits were not regarded as anastomosis. An endoscopic image of an anastomotic leak is shown in (Fig. 1A), and a CT image is shown in (Fig. 1B).

After the diagnosis of anastomotic leak, the patient’s oral intake was stopped, and antibiotic treatment was initiated. In all patients, a stent was placed during endoscopic examination. In patients with suspected anastomotic leak, one of the 3 approaches was applied according to the surgeon’s preference. These approaches include percutaneous drainage by interventional radiology, endoscopic treatment, and endoscopic treatment in patients with persistent leaks despite percutaneous drainage. Patients who were treated only with percutaneous drainage and who did not receive any endoscopic treatment were excluded from the study.

**Figure 2.**
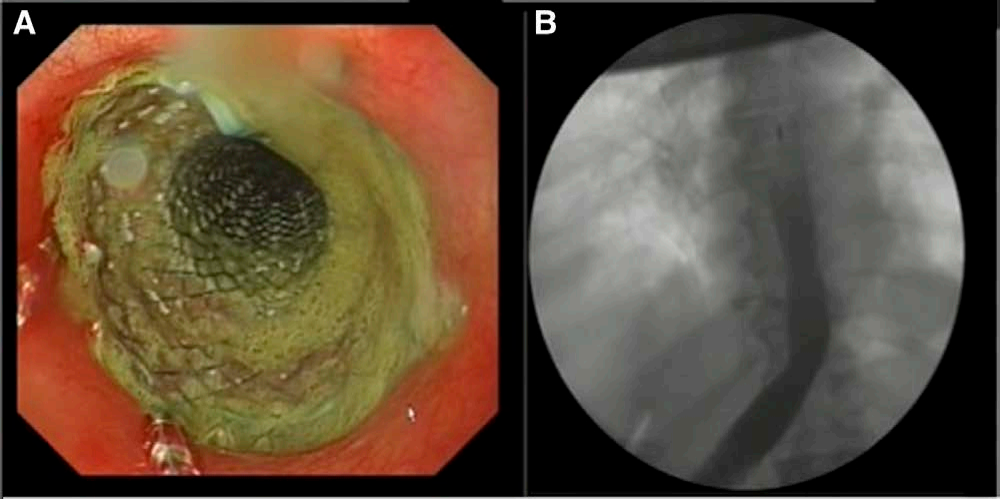
Endoscopic and radioscopic images of stent placement.

Upper endoscopy was performed with fluoroscopic assessment while the patients were under propofol anesthesia. CO_2_ insufflation was not performed and the patient did not undergo tracheal intubation. Micro-Tech (Nanjing Micro-Tech Medical Company, Nanjing, China) or Taewoong Niti-S esophageal FCSEMSs (Taewoong Medical Company, Gyeonggi-do, South Korea) with a length of 60 to 160 mm and shaft diameter of 20 to 22 mm, with double-step shoulder ends, were used. Under fluoroscopic guidance, a metal marker was used to mark the leak location and the guidewire was left distal to the leak. The endoscope was removed because the metal stents were unsuitable for passage through the endoscope. A metal stent was inserted over the guidewire under fluoroscopic guidance with the metal marker displaying the anastomosis line in the middle of the stent. The proximal end of the stent was fastened to the esophageal mucosa using 2 to 4 hemoclips to prevent distal migration. In patients with suspected leak, an X-ray was performed within the first hour after the end of the procedure. The procedure was repeated immediately if stent migration was detected on radiography or if clinical signs of anastomotic leakage were present. Endoscopic and radioscopic images of the stent placement are shown in (Fig. 2A and B).

Endoscopic reassessment was performed 4 to 8 weeks after the first procedure in the patients who showed clinical improvement. The stents were removed by holding the suture at the proximal end of the stent using forceps under endoscopic guidance. If the suture could not be grasped, the stent was removed by grasping its proximal portion. After removal of the stent, endoscopy and CT using an oral contrast agent were performed to evaluate whether there was a leak, and the placement of a subsequent stent was performed in cases of a persistent leak. The permanent drains were removed approximately 7 to 10 days after it was proven that there was no leakage or persistent infection. Oral feeding was not given to patients who underwent endoscopic treatment until the anastomotic leak was healed (until FCSEMS was removed). In order to reduce the risk of aspiration, a naso-enteral tube was placed during the procedure in patients with advanced age and confusion. After clinical stability, a decrease in inflammatory markers, and the reduced surgical drainage, all patients were given enteral nutrition by inserting a naso-jejunal tube.

### 2.4. Technical success assessment

The technical success of the procedure was considered to be the correct endoscopic placement of the FCSEMS by accurately passing the anastomotic leak, and it was assessed by performing endoscopic reevaluation and chest X-ray imaging soon after stent placement.

### 2.5. Clinical success assessment

Clinical success was defined as the complete cessation of anastomotic leak as a result of FCSEMS usage in the correct and appropriate place and position according to the results of endoscopic reevaluation and chest radiography evaluations immediately after stent removal and without any requirement for additional invasive interventions such as surgery, percutaneous drainage, or both. Procedure failure was defined as the continuation of the leak despite the placement of a single stent or multiple consecutive endoscopic stents (a new stent when there was no complete leak closure after the removal of the first stent), need for surgical treatment, death before complete recovery, or need for other methods in addition to endoscopic stent treatment.^[11]^

### 2.6. Ethics

This study was performed in accordance with the ethical guidelines of the 1975 Declaration of Helsinki, modified in 2008. Written informed consent was not obtained from the patients who participated in this retrospective study. This study was approved by the Ethics Committee of Ankara City Hospital (approval no. E1/1809/2021).

### 2.7. Statistical analysis

Statistical analyses were performed using the SPSS 23.0 (IBM SPSS Inc., Chicago, IL) statistical software package. Normally distributed quantitative variables were analyzed using mean and standard deviation values. The median and range values were calculated for the non-normally distributed variables. Qualitative variables are presented as frequencies and percentages. Statistical significance was set at *P* < .05.

## 3. Results

A total of 24 patients (12 of whom were male) were included in the study. The median age of the patients was 60 years (min-max: 38–84 years). All patients underwent surgery for a malignant diagnosis. Fifteen patients had gastric adenocarcinoma, and 4 patients had esophageal adenocarcinoma. The demographic and tumor-related characteristics of the patients are presented in Table 1.

**Table 1:** Demographic and tumor-related characteristics of the patients.

Median age (min-max), years	60.2 (38–94)
Sex (male/female), n	21/3
Type of tumor	
Gastric adenocarcinoma	
(Intestinal/diffuse type)	15 (13/2)
NET	1
GIST	1
Esophageal adenocarcinoma	4
Esophageal squamous cell carcinoma	3
Tumor location	
Cardia	7
Corpus	9
Antrum	1
Mid-esophagus	3
Distal esophagus	4

GIST = gastrointestinal stromal tumor, NET = neuroendocrine tumor, SCC = squamous cell carcinoma.

Twenty-two of the patients had open surgery, while 2 patients were operated laparoscopically. In addition, 19 of the patients had esophagojejunostomy, while 5 of them had an esophagogastrostomy operation. In the esophagojejunostomy group, total gastrectomy was performed in 11 patients, total gastrectomy and splenectomy in 3 patients, subtotal esophagectomy and total gastrectomy in 3 patients, and subtotal esophagectomy-total gastrectomy and splenectomy operations in the remaining 2 patients. Trans-thoracic esophagectomy was performed in 3 of the patients in the esophagogastrostomy group, while transhiatal esophagectomy was performed in 2 patients.

Fever, dyspnea, and abdominal pain were the most frequent symptoms. A CT scan was performed on 17 patients due to the presence of postoperative symptoms such as fever, dyspnea, abdominal pain, pus coming from the drain, pneumonic infiltration, or pleural effusion on chest X-ray. Perianastomotic fluid air and/or leaks were detected in 10 patients. For 5 patients, CT was not needed because of the gastrointestinal system content in the drain or methylene blue coming from the drain. The average time between the operation and the diagnosis of leak was 10.6 days (1–67 days), while the average time between the operation and the endoscopic procedure was 18.4 days (3–67 days). The symptoms and diagnostic findings of patients are shown in Table 2.

**Table 2:** Symptoms and diagnostic findings of the patients.

Symptoms (n = 22)	
Dyspnea	8
Fever and dyspnea	5
Fever	5
Fever and abdominal pain	4
Asymptomatic	2
CT findings (n = 17)	
Perianastomotic air, collection, leak	10
Pneumonia	2
Subdiaphragmatic abscess	4
Intrabdominal free fluid	1
GIS content or methylene blue coming from the drain	5
Leak in esophagography	2
Elevated WBC and/or CRP	22

CRP = C-reactive protein, CT = computed tomography, GIS = gastrointestinal system, WBC = white blood cell count.

**Figure 3.**
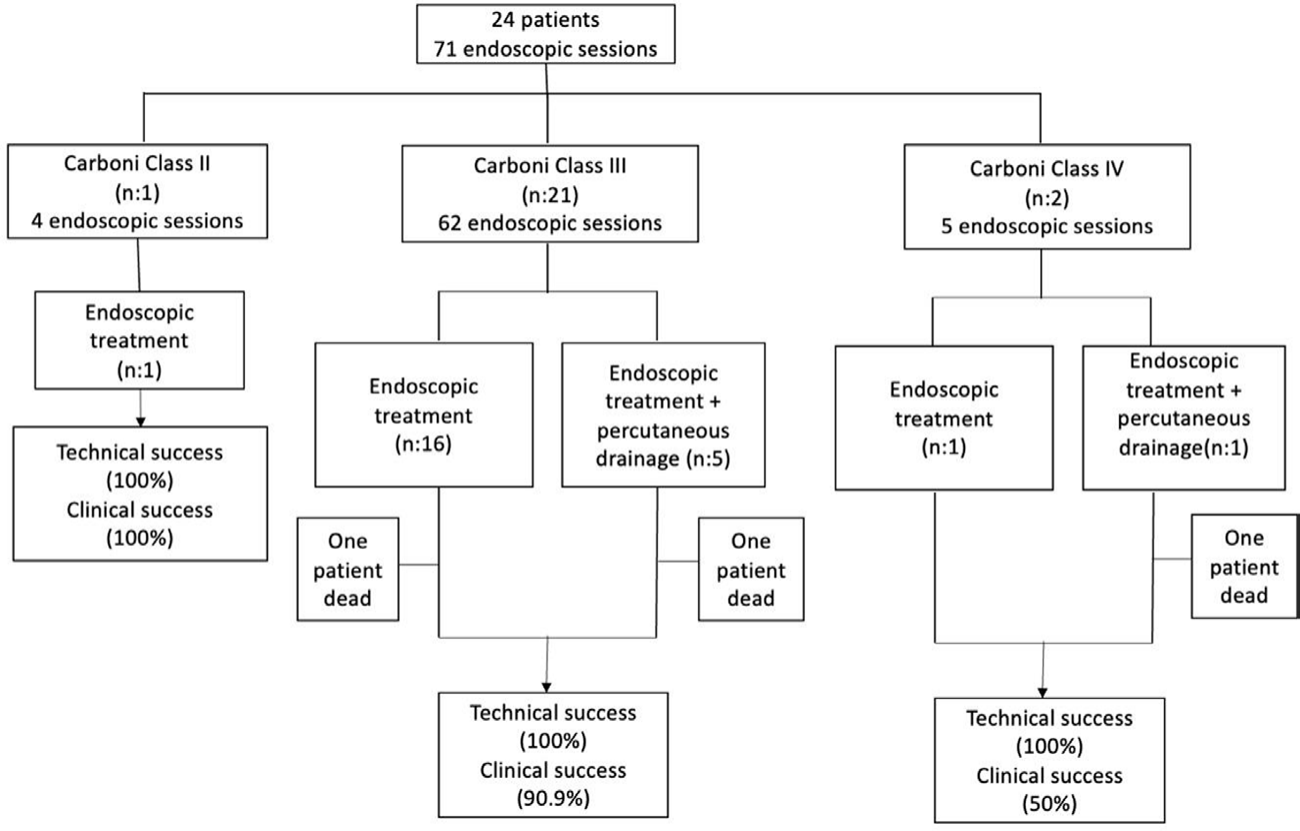
Technical and clinical outcomes of the procedures according to the Carboni classification.

According to the Carboni classification, 1 patient was a class II case, 21 patients were class III, and 2 patients were class IV. Eighteen patients underwent endoscopic stent treatment, while 6 underwent both percutaneous drainage by interventional radiology and endoscopic treatment. In 6 patients, endoscopic procedures were performed in 6 patients due to the persistence of leak findings despite percutaneous drainage before the endoscopic procedure. A naso-enteral tube was placed during the procedure in 7 patients with a high risk of aspiration. A total of 71 endoscopic sessions, with a median number of 2.9 (1–5) procedures, were performed on 24 patients. In total, 37 stents were used in this study. Thirteen patients needed 1 stent each (54.1%), 9 patients needed 2 stents each (37.5%), and 2 patients needed 3 stents each (8.4%). No significant correlation was found between the stage of anastomotic leakage according to the Carboni classification and the number of stents used. Patients with less anastomotic leak were found to have undergone more procedures than those with more leaks. The reason for this was the misconception caused by the low number of patients who were class II or class IV. The technical and clinical outcomes of the procedures, according to the Carboni classification, are shown in (Fig. 3).

The overall clinical success rate was 87.5% (21/24). While the clinical success rate was 94.7% (18/19) among patients with esophagojejunal anastomosis, this rate was 60% (3/5) among patients with esophagogastric anastomosis. Three patients died due to failure of endoscopic treatment. Of these patients, 2 died from pulmonary infection and 1 died from mediastinitis (12.5%).

Complications related to stent placement were observed in of 50%. The most common complication was stent migration, occurring at a frequency of 37.5%. Stent migration occurred in 2 patients with esophagogastric anastomotic leak and 7 patients with esophagojejunal anastomotic leak, and the difference between the rates of stent migration in these 2 groups of patients was not statistically significant (40% vs 37%, *P* = .52). Bleeding occurred in 1 patient, tracheoesophageal fistula occurred in 1, and stent-associated polypoid lesion developed in another. The median retention time of the stents was 68.8 (26–190) days, and the median hospital stay of the patients was 62.7 (24–145) days. The median follow-up period was 11.4 (2–37) months. The post-procedural outcomes and follow-up details of the patients are presented in Table 3.

**Table 3:** Post-procedural outcomes and follow-up details of the patients.

	n	%
Total number of cases with clinical success	21/24	87.5
Esophagojejunal anastomosis success	18/19	94.7
Esophagogastric anastomosis success	3/5	60
Median retention time of stent (d)	68.8	26–190
Median length of hospitalization (d)	62.7	24–145
Complications	12/24	50
Stent migration	9	37.5
Bleeding	1	4.2
Tracheoesophageal fistula	1	4.2
Stent-associated polypoid lesion	1	4.2
Follow-up (mo)	11.4	2–37
Mortality due to anastomosis leak (n)	3	12.5

## 4. Discussion

We reported 24 patients who underwent endoscopic placement of FCSEMSs for esophageal leaks due to cancer surgery, with a technical success rate of 100% and a clinical success rate of 87.5%.

Appropriate treatment of infection, elimination of contamination, and endoscopic FCSEMS placement play an essential role in the treatment of anastomotic leaks. While surgical revision is accepted as the conventional protocol for the treatment of leaks, various conservative treatment protocols, including endoscopic stent placement, have been reported in the literature with different clinical success rates.^[4,12–14]^ In recent studies, different types of stents have been placed for the closure of leaks that occurred due to benign and malignant etiologies, and other results have been obtained with clinical success rates between 59% and 78%.^[15–17]^ Hoeppner et al^[6]^ included 35 patients with esophageal anastomotic leaks in their long-term study. Of these patients, 30 had underlying malignant diseases and 5 had underlying benign disorders. Five of these patients underwent surgery, and SEMS procedures were performed in 30 patients in total. A total of 48 stents, 32 partial and 16 FCSEMSs, were placed in the patients. Anastomotic leak sealing was achieved in 24 patients (69%). When partially and fully covered stents were compared, no significant differences were observed in terms of complications.

In a study conducted by Boeckel et al,^[16]^ 17 patients with benign esophageal rupture and 32 patients with postoperative esophageal anastomosis leaks from 3 different centers were evaluated. Eighty-three fully and partially covered stents were used in this study. The clinical success rate was 76%. No significant difference in clinical success was found between fully covered and partially covered stents.

Although the clinical success rate in our study was 94.7% among patients with esophagojejunal anastomosis leaks, the success rate in those with esophagogastric anastomosis leaks was 60%. The higher clinical success rate in patients with esophagojejunal anastomosis leaks than in patients with esophagogastric anastomosis leaks in our study was similar to the results of other studies.^[6,15]^ This could be because the esophagus and jejunum have similar lumen diameters.

In our study, the complication rate of endoscopic FCSEMS placement was similar to that reported in most other studies (24–46%).^[6,16,18–20]^ Various complications, such as stent dislocation, gastrointestinal bleeding, perforation, or intestinal-tracheal fistulas, may occur in the process of esophageal stenting.^[4,21]^ In our study, stent complications were seen in 50% of the patients, and the most common complication was stent migration, with an incidence of 37.5%. Stent migration occurred in 2 patients with esophagogastric anastomotic leaks and 7 patients with esophagojejunal anastomotic leaks. These disproportionate numbers may have been observed because of the small number of patients in the esophagogastric anastomosis leak group. The stents were appropriately repositioned in all the patients. The stent migration rate was similar to that reported in previous studies (19.2–29%).^[6,16]^ Mild bleeding occurred in 1 patient and was stopped with conservative treatment. One patient with a tracheoesophageal fistula was treated with an FCSEMS, and a stent-associated polypoid lesion occurred in 1 patient. No serious bleeding or esophageal rupture developed during the procedures. These results confirm that endoscopic FCSEMS placement could be recommended as a safe technical procedure for esophageal leaks.

The median time between the operation and detection of leaks in our study was 10.6 days (1–67 days), whereas the median time between the operation and the endoscopic procedure was 18.4 days (3–67 days). The reasons for the delay between the detection of the leak and the procedure were the time it took to locate the stent, the time adjustments required in the patients who were taking anticoagulants, and waiting for the effect of the procedure in patients with percutaneous drainage. As we included patients who underwent endoscopic treatment, we did not include patients who underwent percutaneous drainage but did not receive endoscopic treatment. While information in the relevant literature shows that esophageal stents are removed after a median time of 2.4 months (1.5–10.5 months), the follow-up period for patients with esophageal stents has been reported to be as long as 6.9 months (0.8–147.6 months).^[22,23]^ In our study, the stents were removed after a median time of 2.3 months (0.9–6.3 months), and the median patient follow-up time was 11.4 months (2–37 months). The median length of hospital stay among patients in our study was 62.7 days (24–145 days). The stent follow-up times in our study and those of other studies were similar.^[22,23]^

Esophagogastric and esophagojejunal anastomotic leaks are serious life-threatening complications with high mortality rates after surgical revision.^[2,4]^ The endoscopic placement of FCSEMSs has emerged as an alternative treatment with reported mortality rates of 0% to 33%.^[6,15]^ The mortality rate in our study was 12.5% and similar to those found in other studies. Compared to the results of our study, high mortality rates in the range of 33% to 64% due to revision surgery for anastomotic leak after upper gastrointestinal reconstruction have been reported in previous studies.^[2,24]^ Therefore, endoscopic FCSEMS placement should be considered among patients with an esophageal leak due to cancer surgery.

A recent multicenter study evaluated the efficacy of endoscopic treatment of postsurgical leaks. In this study, which included 206 patients who underwent sleeve gastrectomy, gastrectomy, and esophagectomy, the success rate of endoscopic treatment and adverse events were evaluated, and an endoscopic leak closure rate of 80.1% was observed with a median follow-up duration of 52 days. Side effects were observed in 39.3% of patients, and the rate of mortality due to the procedure was reported to be 12.4%.^[25]^ Although our study included a small number of patients, it was standardized because all patients had undergone surgery for malignancy, it was a single-center study, and all endoscopic procedures were performed by the same endoscopist. The success rates, adverse events, and mortality rates in our study and the aforementioned multicenter study were similar. These results confirmed that endoscopic FCSEMS placement could be considered one of the primary treatment protocols for esophageal leaks.

Our study had some limitations. First, the retrospective nature of this study could be a limitation. Because anastomotic leaks are unpredictable and uncommon, designing a prospective study with a large number of patients is extremely difficult. Second, not only were the FCSEMS types and sizes different among the patients, but the stent removal time intervals, concurrent additional treatment protocols, and procedure timings were also different. These distinctions are significant factors that can influence leak closure, complication formation, and clinical success rate. In light of the lack of standardization in the procedures, we attempted to address this issue by standardizing the study to include all consecutive patients who received endoscopically placed FCSEMSs in procedures performed by the same endoscopist.

## 5. Conclusion

In conclusion, endoscopic placement of an FCSEMS is a safe and effective treatment for esophagojejunal and esophagogastric anastomotic leaks.

## Author contributions

**Conceptualization:** Mustafa Cengiz, Bulent Odemis, Muhammed Bahattin Durak.

**Data curation:** Mustafa Cengiz, Bulent Odemis, Muhammed Bahattin Durak.

**Formal analysis:** Mustafa Cengiz, Bulent Odemis, Muhammed Bahattin Durak.

**Investigation:** Mustafa Cengiz, Bulent Odemis, Muhammed Bahattin Durak.

**Methodology:** Mustafa Cengiz, Bulent Odemis, Muhammed Bahattin Durak.

**Project administration:** Mustafa Cengiz, Bulent Odemis, Muhammed Bahattin Durak.

**Resources:** Mustafa Cengiz, Bulent Odemis, Muhammed Bahattin Durak.

**Software:** Mustafa Cengiz, Bulent Odemis, Muhammed Bahattin Durak.

**Supervision:** Mustafa Cengiz, Bulent Odemis, Muhammed Bahattin Durak.

**Validation:** Mustafa Cengiz, Bulent Odemis, Muhammed Bahattin Durak.

**Visualization:** Mustafa Cengiz, Bulent Odemis, Muhammed Bahattin Durak.

**Writing – original draft:** Mustafa Cengiz, Bulent Odemis, Muhammed Bahattin Durak.

**Writing – review & editing:** Mustafa Cengiz, Bulent Odemis, Muhammed Bahattin Durak.
